# Quick fabrication of 3D-printed prosthetic sockets with embedded sensors to track limb motion

**DOI:** 10.3389/fresc.2026.1805115

**Published:** 2026-09-21

**Authors:** Joan E. Sanders, Amy P. Culter, Mathew J. Weissinger, Paul Fallon, Joseph C. Mertens, Katheryn J. Allyn, Benjamin N. Sanders, Joseph L. Garbini

**Affiliations:** 1 Department of Bioengineering, Sanders’ Research Laboratory, University of Washington, Seattle, WA, United States; 2 Department of Mechanical Engineering, University of Washington, Seattle, WA, United States

**Keywords:** 3D printing, activity tracking, amputee, CAD/CAM, gait analysis, limb health, patient awareness, prosthesis

## Abstract

**Introduction:**

A simple time-efficient method to fabricate prosthetic sockets with embedded sensors to track limb motion may facilitate the clinical application of continuously collected quantitative data in prosthetics practice.

**Methods:**

A 3D-printed inner structure of the socket shape with debosses on the outside surface to hold sensors was fabricated, the sensors were affixed in place, and the socket was laminated and trimmed.

**Results:**

The time to fabricate an embedded sensor socket was 12% less than that required to fabricate a traditional carbon-fiber socket with sensors. The investigational sockets increased in volume slightly, <0.2%, over the course of 4 weeks of take-home use, likely because of compression of the inner structure material during ambulation. Sockets with a vapor-smoothed finish had a significantly lower coefficient of friction (COF) with the liner fabric backing than sockets with a normal surface finish (*P* < 0.01), though participant preference for the lower COF socket was mixed.

**Discussion:**

The speed, quality, and durability of the investigational sockets warrant clinical studies to determine which quantitative limb motion metrics (e.g., pistoning, volume, anterior distal motion) help prosthetists meaningfully enhance their patient care.

## 1 Introduction

The limb-socket interface is vital to the prosthesis user as it is the site of relative motion between the residual limb and socket and stress transfer between the prosthesis and the person with limb amputation. The motion and stress at the interface dictate the socket fit, which in turn affects comfort, gait stability, and limb health. Socket fit is the most important issue affecting prosthesis users' satisfaction with their prosthesis (1, 2). Potentially, prosthetists could use the data from limb-socket interface sensors to help decide their patients' socket design, for example its shape, material, and suspension. Data from continuous monitoring could be used to help identify the cause of patients' injuries and provide insight into how to avoid them in the future (3, 4). Sensor data may facilitate insurance justification and in the long term may provide such valuable data to prosthetists and prosthesis users that it receives L-coding to cover the sensored-socket fabrication costs.

Numerous socket interface stress sensing technologies have been pursued (5, 6) including thin piezoresistive sensors, capacitive sensors, strain-gage sensors, load cells, MEMS pressure sensors, vacuum sensors and bubble sensors. Most of these sensors the prosthetist applies to the residual limb or to the inside of the socket after it is fabricated. This methodology may be practical for in-clinic use or short-term monitoring, but maintaining functionality during long-term monitoring is difficult, in part because of damage to the sensing elements. Researchers have drilled holes in sockets to support and protect pressure sensors (7, 8) but this process may restrict sensor placement to relatively flat locations, limiting versatility and the clinical utility of the data. It may also extend the time to fabricate the socket.

Tracking motion instead of pressure offers advantages in sensor durability because motion sensing does not require contact between the socket and residual limb/liner system. Motion sensors may be embedded beneath the surface, well protected from mechanical damage (9–11). Another advantage is that motion sensing provides insight into limb-socket interaction during swing phase, not just stance phase. Imaging and motion capture systems and proximity sensors have shown promising results to track soft tissue pistoning during ambulation, suggesting that swing phase is relevant (12–16).

Efforts to fabricate definitive 3D printed sockets have progressed over the past decade. In general, benefits to cost-effectiveness, ease of customization and accessibility have been reported. For example, in lower limb prosthetics 3D printed sockets have shown enhancements in comfort, gait parameters, and customization, particularly in amphibious and partial foot prostheses (17–19).

The use of sophisticated engineering design strategies, e.g., design guided by finite element model simulation, and advanced fabrication techniques like multi-jet fusion have enabled definitive sockets to be commercially printed for patient use. Reinforced 3D printed sockets have been pursued where after an inner layer was printed from a weak material such as polylactic acid (PLA), the outer surface was reinforced using stronger materials such as cement, carbon fiber, carbon-Kevlar or fiberglass (20). In the future the reinforcing materials may be used to integrate componentry such as pin locks, volume adjustment systems and vacuum systems. Some components depended on woven carbon fiber and resin to firmly bind them to the socket. It was suggested that sensing systems may similarly benefit from integration before the 3D print is reinforced. This hybrid manufacturing approach would allow conventional electronic components to be integrated into the socket wall. As Lehmhus et al (21). described, the integration of sensors into 3D printed parts in automated manufacturing can be accomplished through “hybrid” processes. A single manufacturing system may combine several different processes, or a single process may have enough flexibility to create the whole component. The authors noted, however, that electronic components printed using current additive manufacturing systems have not achieved as effective performance as conventional strategies where electronic systems were placed within the print. Efforts have been made to embed sensors in soft systems to measure pressure, temperature and strain including weaving them into textiles or encapsulating them into elastomers (22, 23). Descriptions of lamination methods to place sensors to detect stress and strain in composites have been presented in structural health monitoring, e.g., those by Javidinejad (24) and Weck (25). The methods presented in the present research sidestep challenges in composite property degradation experience by others incorporating the sensors into the matrix because the sensors lie between the 3D print and carbon fiber lamination rather than within the lamination.

The purpose of this research is to create and test a method for quick and reliable fabrication of 3D printed sockets with embedded sensors for limb motion tracking. Our objective is a robust strategy that consistently produces high-resolution socket shapes and functional sensors, is more time-efficient to fabricate, and is less prone to manufacturing error than a traditional socket layup. The system is designed to produce embedded sensor sockets that function seamlessly with the prosthesis and prosthesis user and do not require maintenance.

## 2 Methods

A three-step process was executed to make the embedded sensor sockets. In the first step, the *inner structure* of the socket was prepared and 3D-printed (Figure 1A). In the second step, sensor antennae, electronics, lead wires and componentry were placed in debosses on the outside surface of the inner structure (Figure 1B). In the third step, a carbon-fiber lamination was applied to add structural integrity, and the brim was trimmed (Figure 1C).

**Figure 1:**
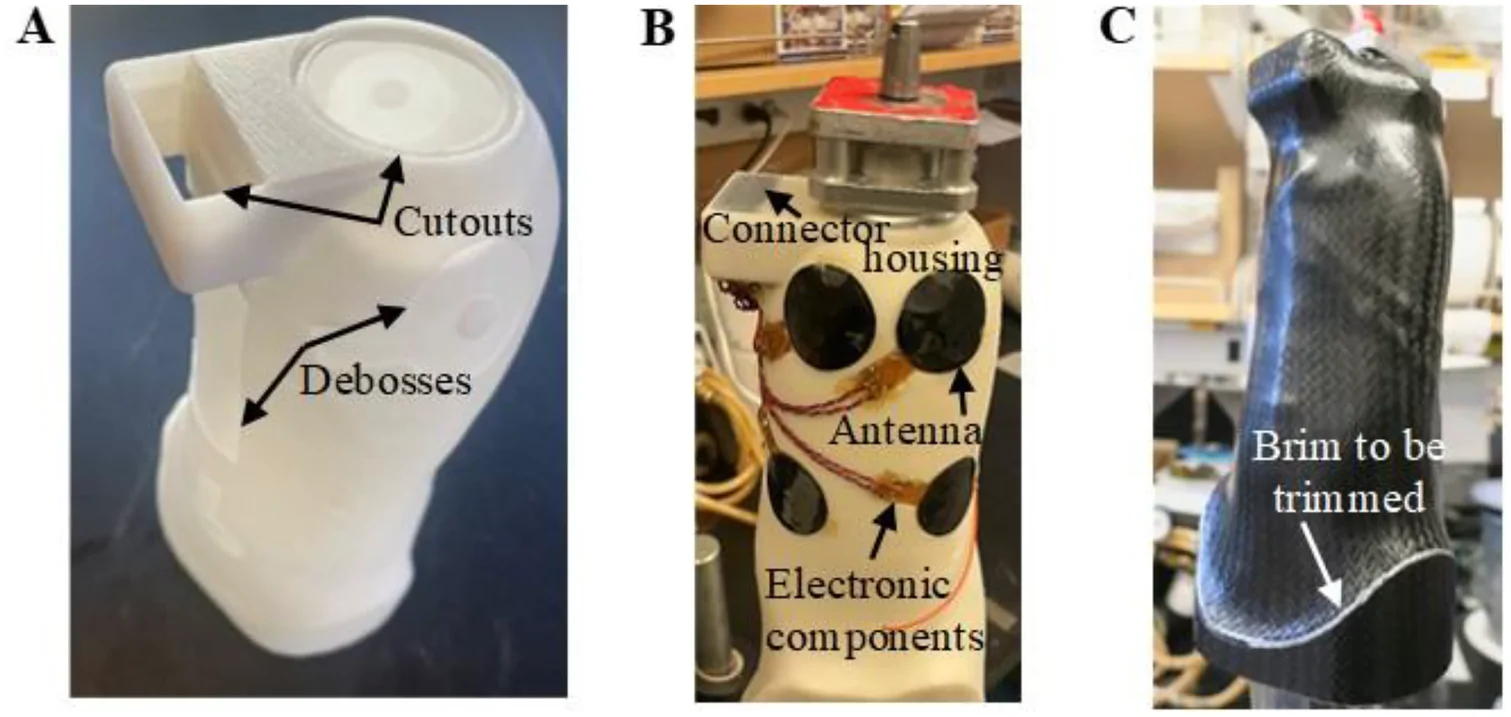
**(A–C)** three-step process for embedded sensor socket fabrication. **(A)** Step 1: Prepare the inner structure. Cutouts for the connector and 4-bolt adapter and debosses for the sensor antennae and wires are shown; **(B)** Step 2: Place the componentry and sensing system onto the outside of the inner structure; **(C)** Step 3: Laminate and trim.

### 2.1 Material selection

Materials commonly used in 3D printed prosthetic socket research include PLA, polycarbonate, polypropylene (PP), polyamide 12 and carbon fiber reinforced composites (26, 27). The materials listed in Table 1 were considered for fabrication of the inner structure. This list was generated from materials available through candidate vendors capable of producing 3D printed shapes of length at least 40 cm, our maximum expected socket length, in their facilities. Multi-jet fusion (MJF) materials were not included because the available facilities did not meet the minimum length requirement. The material's glass transition temperature needed to be above 104 °C to tolerate fabrication of the carbon fiber layup in step 3 (Figure 1C). The Young's modulus and tensile strength needed to be comparable to the Nyglass material currently used in traditional carbon fiber layup fabrication techniques (28). We estimated a Young's modulus of at least 3 GPa and a tensile strength of at least 34 MPa were necessary.

**Table 1:** Materials considered for fabrication of the inner structure.

Material	Method	Description	E (GPa)	TS (MPa)	HDT (°C)	Cost/kg
X Ceramic 3280	LSPC	Rigid ceramic composite, chemically resistant and high Young's modulus; wind tunnel use	9.4	40	280	$300
X Peek 147	LSPC	High temperature PAEK thermoplastic, strong and rigid similar to ABS but higher HDT	3.2	75	230	$380
Accura: Bluestone	SLA	Strong rigid plastic, high water resistance, composite of unlisted ceramic mixture; wind tunnel use	7.6	66	267	$250
Somos PerFORM*	SLA	Rigid ceramic composite, UV cured then can be thermal post cured, becomes stronger but less stiff	*10*.*5*	*75*	268	$440
Form Rigid 10 k (w/, w/out post cure)	SLA	Glass embedded stiff resin, higher E/lower TS when post cured	9.0, 10.0	65, 58	218	$185
Blended Nylon 12's:						
- Carbon Filled (PA 602-CF)	SLS	Strongest, most rigid, lightest nylon blend; used in sports equipment, race car parts	5.0	85	179	$160
- Glass Filled	SLS	Similarly rigid to carbon filled but weaker, heavier; less expensive; used for tooling	5.9	38	179	$110
- HST	SLS	Slightly weaker than others but better thermal resistance and denser structure; infused with mineral blend	3.0	34	184	$150

* Previously used by Sanders’ lab to make an instrumented distal insert (13).

LSPC, lubricant sublayer photo-curing; SLA, stereolithography; SLS, selective laser sintering; E, Young's modulus; TS, tensile strength; HDT, heat deflection temperature.

Three materials were selected from Table 1 for further consideration in the following preferential order: glass-filled blended nylon 12 (SLS); carbon-filled blended nylon 12 (SLS); and Somos PerFORM (SLA). The SLS materials were chosen for their price point and ease of processing when transferred to in-house production (no post-cure required). Glass-filled nylon was preferred over carbon-filled nylon because it had comparable stiffness and was less expensive. The Somos PerFORM, an SLA material we used in a recent distal sensor insert research effort (13), was considered a backup if the other two proved not strong enough for our application.

To evaluate the mechanical integrity of the selected materials, we fabricated short hemispherical distal sections of prosthetic sockets of each material. The samples were 45 mm in height and diameter, with a center cutout for a 4-hole locking pin adaptor (Figure 2A). A carbon fiber lamination applied using traditional carbon fiber layup methods was added over each sample. The inside shapes of the short hemispherical distal sections were measured before and after the mechanical tests described below.

**Figure 2:**
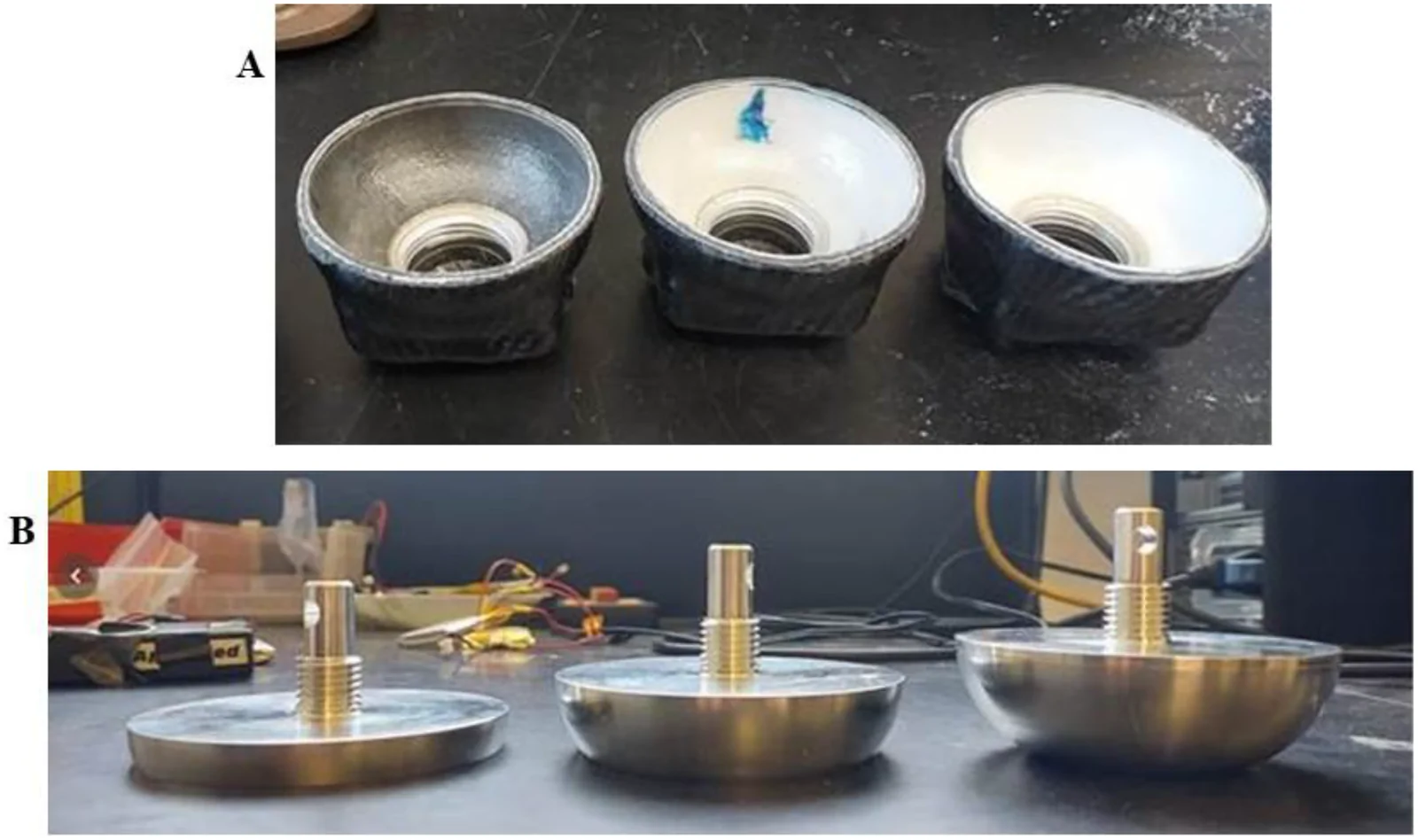
Distal socket section material testing. **(A)** Three hemispherical distal sections prepared for testing. Materials from left to right are carbon-filled blended nylon 12, glass-filled blended nylon 12 and Somos PerFORM. **(B)** Load applicators from left to right of 10-, 20-, and 30 mm height were used. The taller the load applicator, the greater the surface area stressed on the hemispherical distal sections.

We subjected the samples to 100,000 cycles applied at 1.5 Hz (approximately 3,000 steps per day for 33 days) in a uniaxial mechanical testing machine (5,944, Instron) using three different load applicator shapes (Figure 2B), thus a total of 300,000 cycles. The 3,000 daily step count corresponded to the lower end of the activity range for transtibial prosthesis users from a 2021 study (29). Three months of use was considered sufficient time to observe meaningful plastic deformation. The three different load applicators were designed to apply load to the upper quarter, upper half, and full surface of the hemispherical distal section, respectively. The load applicator was backed off the surface 0.5 mm during each cycle to ensure it completely unloaded the sample. A peak force of 200 N was applied, approximately 20% body weight of an average prosthesis user. A 20% body weight was selected since the surface area of the test samples was approximately 20% of the area of a transtibial prosthetic socket. Loads were applied continuously for each 100,000-cycle test, though brief breaks were required three times to start a new data file because of file size limits on the testing machine. A 2.0 mm inner-structure thickness was selected because this was the minimum thickness necessary to form the debosses on the outside surface to support the antennae.

After load application was completed, the hemispherical distal sections were scanned. The shapes from before and after loading were compared using a custom algorithm developed previously (30). The algorithm implemented a combination of global and local shape similarity techniques to align the two shapes. The volume difference between the socket shapes expressed as a percentage of socket volume, the median radial error and the 1st and 3rd interquartile range of radial error were calculated.

### 2.2 Prepare the inner structure

The inner structure was a manufactured part of the socket. Its internal surface was the inside of the socket. Its outside surface was shaped with cutouts and debosses to hold elements of the sensing system.

A digital file of the participant's current socket shape was used as a starting point for the design of the inner structure (Figure 3A). If no digital file was available, an industrial scanner (FARO Arm, Quantum-M, FARO Technologies; accuracy setting 0.02 mm) and software (Geomagic, Design X 64) were used to scan the traditional socket shape using the manufacturer's recommended procedure and create a digital file as described in our prior work (31). Care was taken to ensure the location of the 4-hole adaptor that supported the socket was included in the shape data.

**Figure 3:**
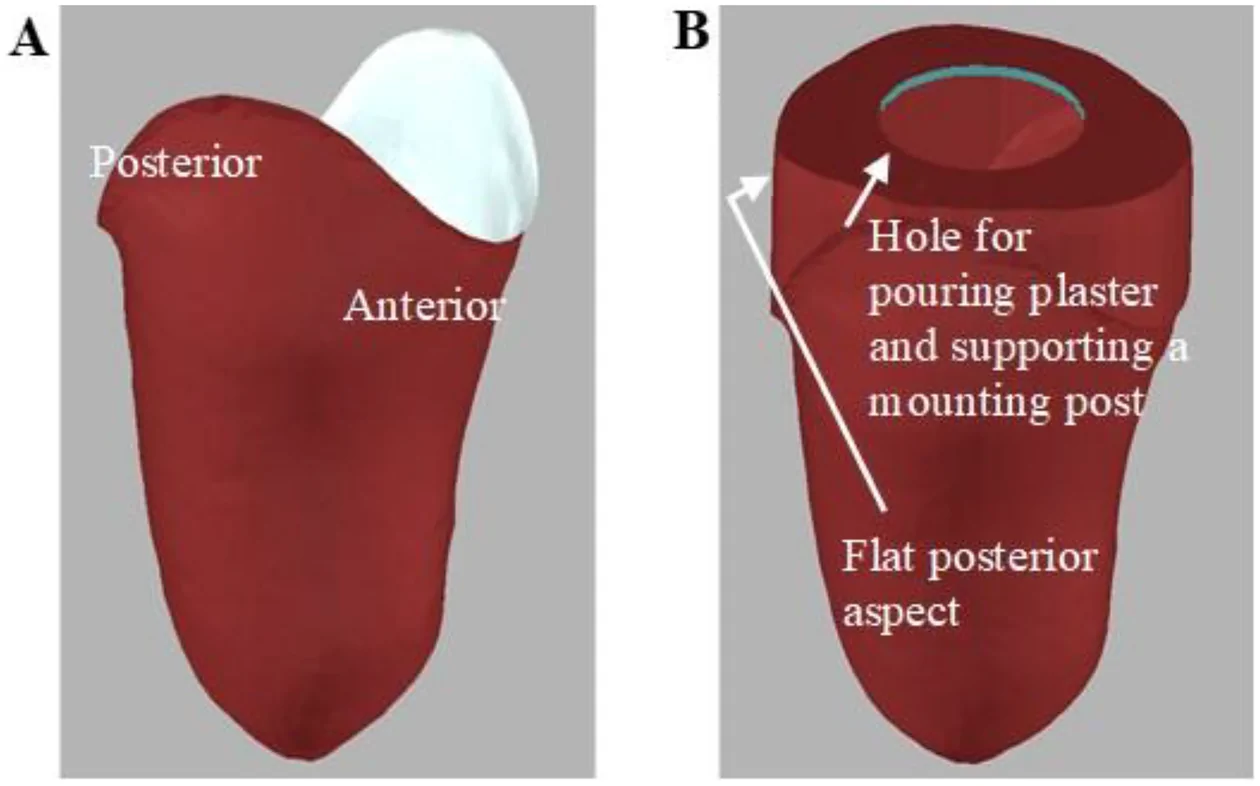
Creating the inner structure. **(A)** Original scanned socket shape. **(B)** The shape from A was outward projected to create a 2.0 mm thick model, the socket shape was extended proximally, and a flat surface at the proximal end with a hole for a mounting post was added. The posterior aspect of the model above the trimline was flat and in this research was used to create a rectangular sample used for friction testing.

The digital file was imported into a computer-aided design (CAD) package (SolidWorks, 2024 Standard Research edition, Dassault Systemes) to create a CAD model. The shape was outward-projected 2.0 mm to create a solid model (Figure 3B). The proximal end of the model was closed, and a hole for the mounting rod was created. A 41 mm diameter hole was placed through the distal end of the socket, centered about the middle of the four-hole adaptor (Figure 4) and in line with the hole on the proximal surface. At the distal end, material was added around this hole to create a lip and support for the 4-hole adaptor. This added material allowed the top of the 4-hole adaptor to line up properly with the model when the technician began the layup in step 3, laminate and trim (described below).

**Figure 4:**
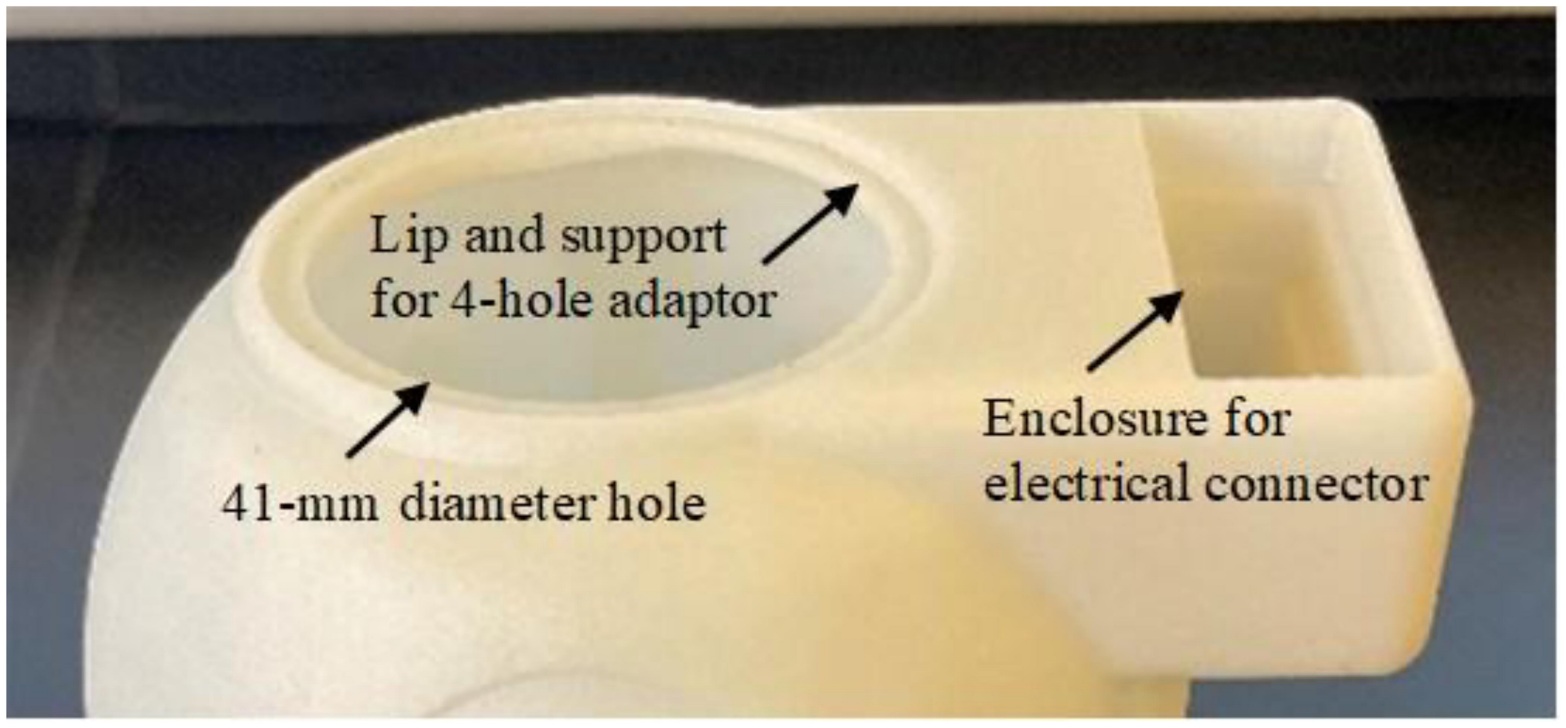
Design of the distal end of the inner structure. A 41 mm diameter hole was designed into the distal end of the socket. A lip and support were added to support the 4-hole adaptor. An enclosure for an electrical connector was added at the right such that its bottom surface was flush with the hole for the adaptor.

A separate model for the electrical connector enclosure was imported into the CAD model. The connector enclosure model was 46.6 mm × 18.3 mm × 22.6 mm and included a cutout so the connector could later be physically mounted into it. The bottom of the connector was in the same plane as the bottom of the socket (Figure 4). The circumferential location of the connector was selected based on convenience of use. We typically placed it on the lateral side. Using the software (*combine* feature), the connector and socket were united so that there was continuous material between them.

Using the software (*wrap* feature), a rectangle was debossed 0.8 mm into the socket above the electrical connector position. This rectangle was 36.6 mm wide and 130 mm tall, though the height could be cut down to 110 mm to accommodate a small socket. The path of the antennae wires into the connector was smoothed to ensure there were no sharp edges that may have damaged the wires.

In prior research efforts, we established the antenna locations based on anatomical markers on the residual limb (11). In the present study, a computer algorithm was written that uses key anatomical features to implement the process described by Ballesteros et al. (11). Alternatively, locations of interest could be selected by the clinical care team and marked on the inside shape of the socket that was subsequently scanned. With either strategy, the antenna location coordinates were added to the CAD model digital file.

A 0.8 mm deep deboss was made at each antenna location (Figure 5). Each deboss was a 9.5 mm ID, 33.5 mm OD annulus. An 11.5 mm wide, 19.0 mm long tail was extended out perpendicular from the center of the annulus in the direction that created the shortest path from the antennae to the connector.

**Figure 5:**
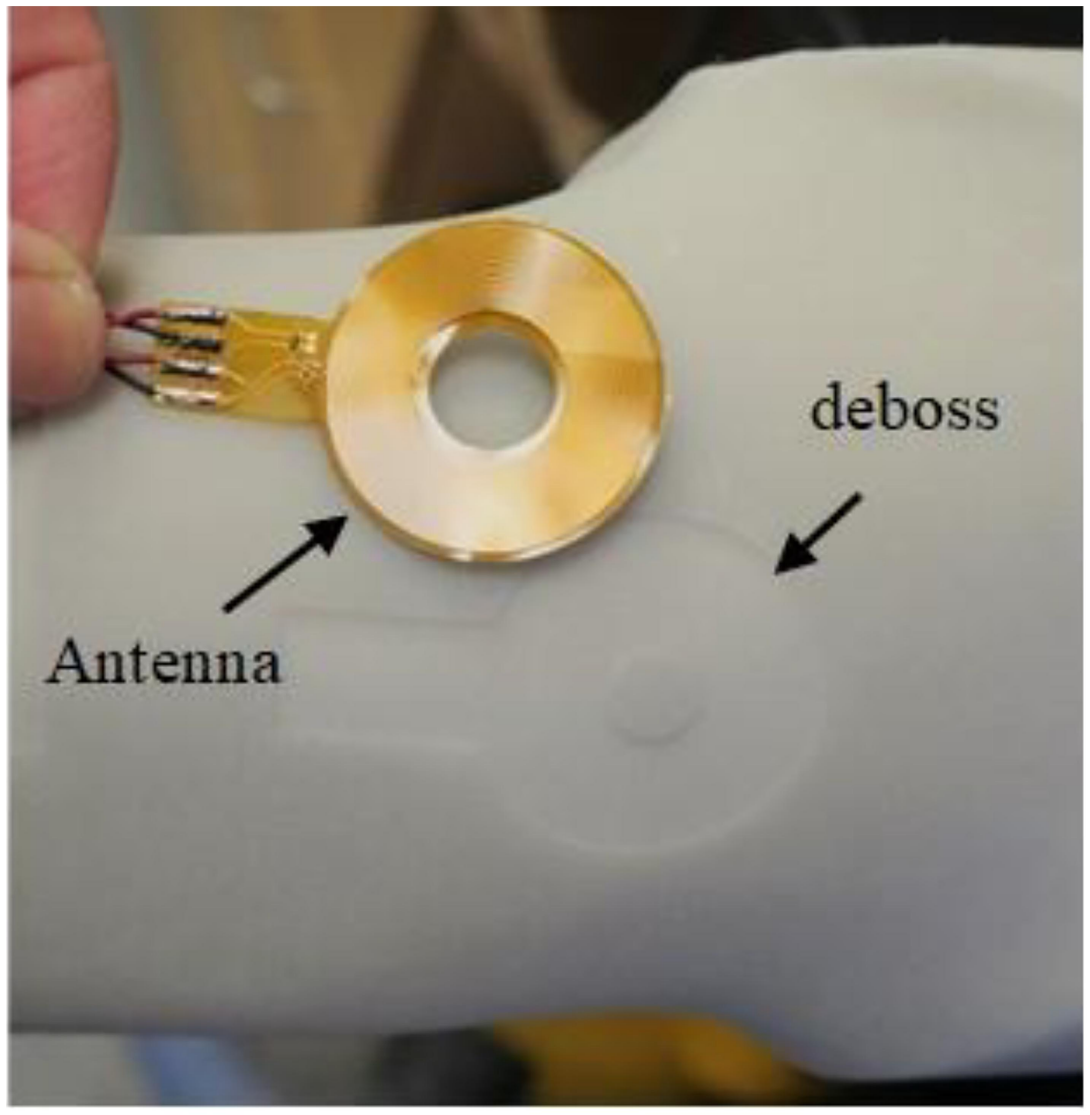
An antenna and deboss are shown.

The antennae, which were 0.15 mm thick, were manufactured by a vendor specializing in printed flexible circuitry. Double-sided tape (SpeedTape, FastCap) cut to the antenna diameter was adhered to the bottom surface of each antenna. During assembly (described below), the protective paper on the opposite side of the tape was removed, and the antennae were adhered into the debosses.

The brim of the CAD model was extended to a surface 6.4 mm above the top edge of the flaps and parallel with the base. The surface was a 2 mm thick plate. A 75 mm diameter hole was placed through the 2 mm thick plate so that the hole connected to the inside of the inner structure (Figure 3B). If the diameter of the proximal brim was less than 75 mm, the diameter was reduced, but in our sockets with embedded sensors fabricated to date, that was not necessary. The hole later simplified pouring plaster into the inner structure to provide mechanical support during fabrication (as described below). The final CAD model was electronically sent to a vendor and 3D-printed. The inner structure replaced and was functionally identical to the initial 2-layer Nyglass lamination of a traditional socket.

### 2.3 Place the socket componentry and the sensing system

The subsequent steps in preparation were similar to techniques used in traditional socket fabrication. The distal end of the 3D printed inner structure had a lip on the external surface and a contour on the inside surface to accommodate the appropriate 4-hole adapter for the prosthesis user's suspension method. For pin suspension we used a shuttle lock (Icelock 674, Össur), and for suction suspension we used a suction plate (LimbLogic 4-Hole Attachment Plate, WillowWood). The technician secured the assembled 4-hole adapter and tooling kit in place with adhesive (+PLUSeries composite 1 min adhesive, Fabtech) (Figure 6).

**Figure 6:**
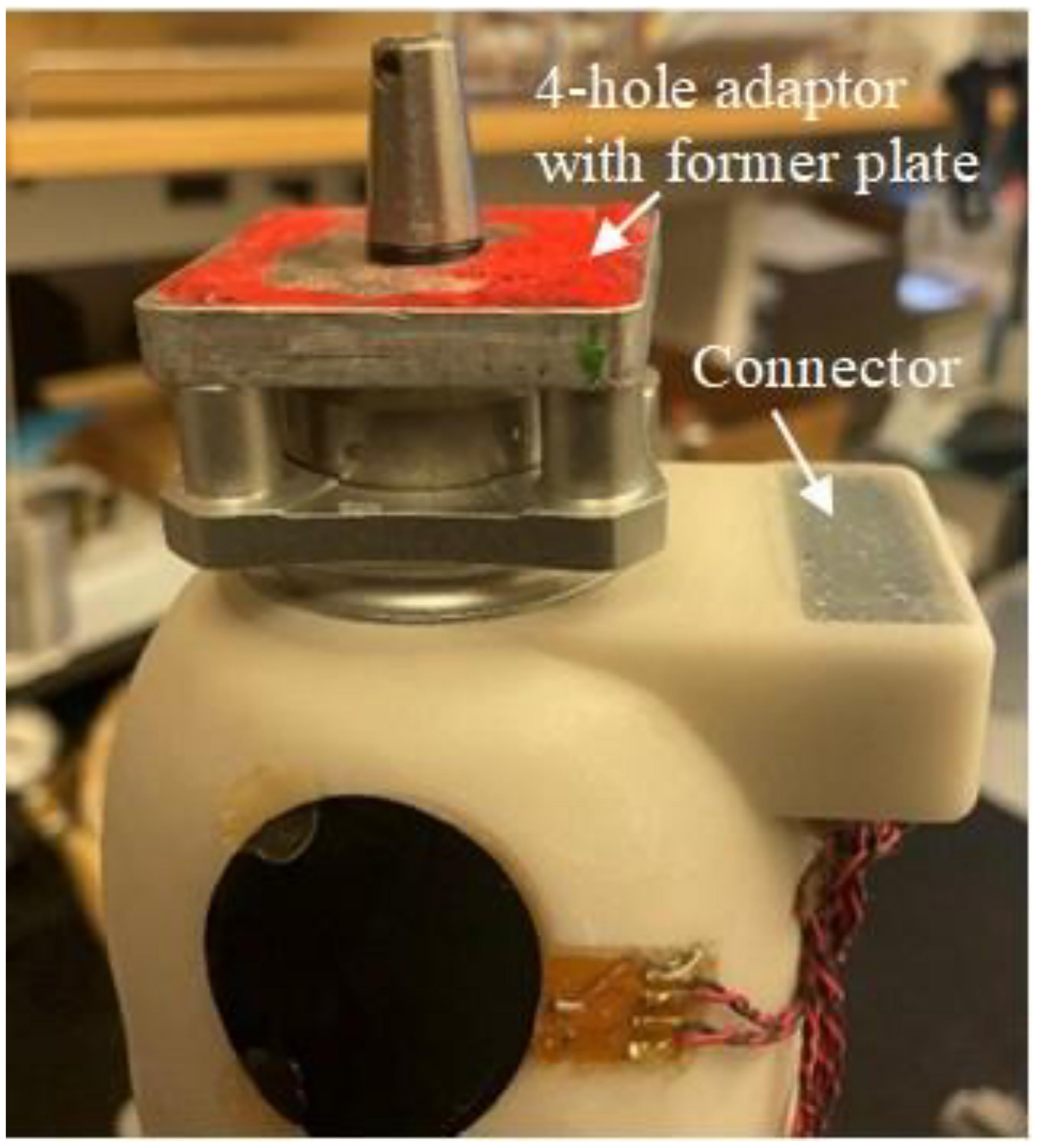
Distal end of the inner structure with componentry in place. The 4-hole adaptor with former plate, one antenna and electronic componentry from the sensor harness, and lead wires into the connector are shown. The top of the connector was potted with adhesive to ensure no resin entered during the lamination process.

In order to prevent deformation under vacuum during the lamination, the 3D printed inner structure was filled with plaster. Similar to the inner lamination of a traditional socket, the 3D-printed outer surface was roughened up using sandpaper to approximately 220 grit to facilitate a strong bond to the outer laminated material.

To expedite the fabrication process, the sensing system components were pre-assembled into one unit (Figure 7A). In this paper, we refer to an assembly of 7 antennae, their attached electronic components (capacitors, thermistors, and lead wires), and their common connector as a *sensor harness*. Sensor harnesses were prepared in bulk in a fabrication process separate from the fabrication of the embedded sensor sockets described here. This practice made the assembly durable and strain relieved the wires, minimizing the risk of damage.

**Figure 7:**
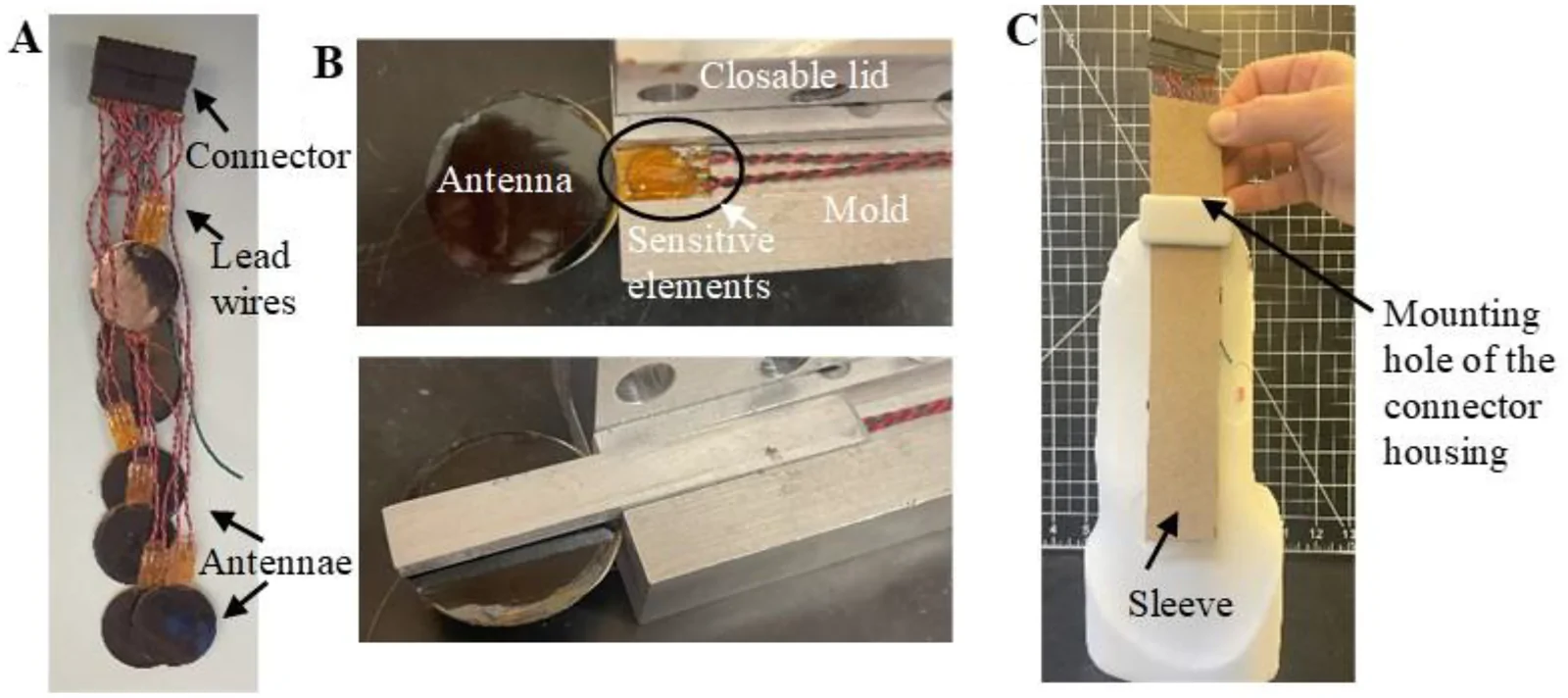
**(A–C)** preparation of a sensor harness. **(A)** A complete sensor harness. The sensor harness included the connector, lead wires, and antennae. **(B)** A mold with a closable lid was used to pot the sensitive elements in hot melt glue. **(C)** The antennae were placed in a sleeve and threaded through the mounting hole of the connector housing.

To prepare a sensor harness, each antenna was placed into a mold (Figure 7B). A 10 mm diameter bolus of hot melt glue was placed at the midpoint of the tab and the lid of the mold was closed to compress the hot melt into an even layer over the electronic components. Ultimately the hot melt acted as an insulative layer, however during the fabrication process its main purpose was to protect the vulnerable electronic components. With the thermistor, capacitor, solder pads, and lead wires encased in hot melt, the risk of mechanical damage was minimized. The lead wires were cut, stripped, crimped, and placed into the connector. All antennae were electrically tested for functionality. The antennae were gathered into a stack and encased in a protective sleeve. The sleeve was used to safely thread the antennae through the connector housing (Figure 7C). Once the connector was fully seated into its housing the protective sleeve was removed.

The lengths of the lead wires were calculated from the CAD model of the inner structure described above to ensure later that the lead wires were not too taut when adhered to the outside surface of the 3D-printed inner structure. A standard wire length for each socket region (proximal, mid-limb, and distal) also proved effective. If necessary, there was room in the crevice next to the connector to bundle excess wire and covered it during lamination.

In locations of high curvature, a hole was punched through the center of the antenna so that it better conformed to the shape (Figure 5). Overlapping wires were avoided to prevent crosstalk between the twisted pairs. The wires were secured with hot melt (Scotch-Weld Hot Melt, 3M) every 5−10 cm as needed to maintain the determined wire route. A ground wire was placed in the carbon fiber mesh in the distal region (Figures 8A,B). The ground wire was necessary because the carbon fiber, which is conductive, otherwise distorted the signals of interest.

**Figure 8:**
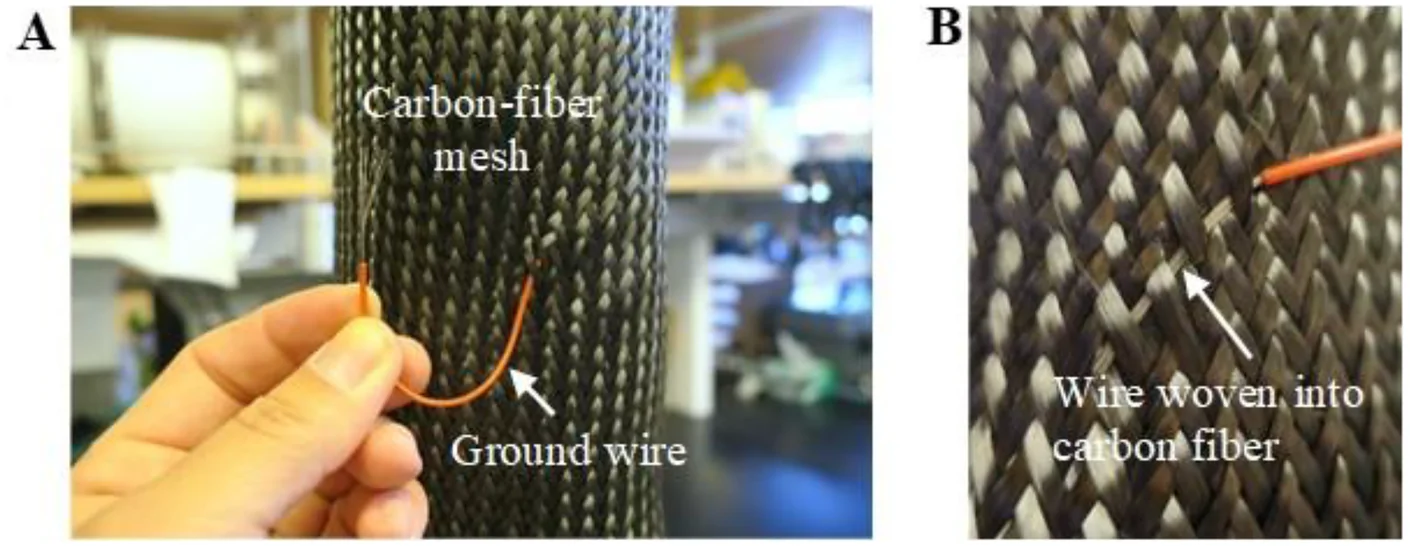
Ground wire. **(A)** A ground wire from the connector was placed in the carbon fiber mesh. **(B)** At least 3 cm of wire was pulled through and woven into the first layer of carbon fiber.

As described in our prior work (11), once the antennae and lead wires were in place, they were tested for functionality. A portable data acquisition system was connected via a serial connection to a PC operating a terminal program (H-term). Each sensor was tested using an approximately 50 mm diameter section of ferrous liner target material (32). When the ferrous liner was covering the antenna the signal values decreased and when the ferrous liner was removed the signal increased. Thermistor function was tested by placing a gloved finger over the thermistor. Warming the thermistor with the finger increased the signal magnitude, and removing the finger cooled it and decreased the magnitude. Improper function of the antennae or thermistor typically was indicative of mechanical failure at a connection point, including the wire crimps and the solder connections for the thermistor, capacitor and lead wires. The sensor harness was repaired if necessary.

Once functionality was confirmed each sensor coil was covered with an adhesive-backed ferrite patch, taking care to ensure the via to the electronic components was exposed. The ferrite provided magnetic and electronic shielding to one side of the antennae The face of the connector plug was potted with a platinum cure silicone (Dragonskin 10 FAST, Smooth On) (Figure 6). The silicone prevented resin from entering the plug during lamination.

### 2.4 Complete the final lamination

The lamination has two purposes—protect the exposed wiring and provide structural integrity to the socket and componentry. The layers of the layup were selected at the discretion of the prosthetist based on the weight and K-level (33) of the participant. We typically used the following layers over the inner structure: nylon, carbon fiber, 2 layers of Nyglass, carbon fiber, nylon. The layers of the layup were cut. Before reflecting the layers back down, the ground wire was pulled through to the first carbon fiber layer. At least 3 cm of exposed ground wire was woven into the carbon fiber tows to maximize contact with the conductive carbon fiber. Carbon fiber tows were also pulled around each of the exposed 4 bolts on the distal end of the connector for a pin suspension socket. The remaining layers were then reflected down and smoothed without introducing wrinkles or bunching. A former plate was placed over the distal end of the 4-hole adapter. The bolt heads were covered with putty for easy removal after lamination.

The rest of the fabrication process proceeded using traditional socket fabrication methods. A wetted PVA bag was pulled over the model, the bottom was sealed, a vacuum (15−20 in Hg) was pulled and the resin was poured into the top of the bag. The layers were saturated, the PVA bag was sealed and the resin was left to cure under vacuum.

Once the resin had fully cured, the socket was finished using a cast saw to remove the excess laminate. The plaster was removed from the inside of the socket, and the brim was sanded using a Trautman Router. The silicone plug was removed once the socket was complete.

### 2.5 Clinical testing

The purpose of the clinical testing was to identify if shape distortion occurred and if so, when did it happen. We also tested how long it took to make the investigational sockets and how heavy they were compared with traditional sockets.

#### 2.5.1 Participants

Participants were included in this study if they were at least 18 years of age, had a transtibial amputation at least 18 months prior, self-report wearing their prosthesis for walking at least 7 h/wk and were deemed by the research prosthetist to be at a Medicare Functional Classification Level (33) of K2 (Had the ability or potential for ambulation with the ability to traverse low-level environmental barriers such as curbs, stairs, or uneven surfaces; typical of the limited community ambulator) or higher. Their limb length needed to be at least 9.0 cm and they needed to be capable of sitting, standing, walking continuously with their prosthesis for at least 2 min and stepping up 5.0 cm onto the surface of a treadmill. Participants were excluded if they were currently experiencing skin breakdown injury or used walking aides (e.g., cane, walker).

#### 2.5.2 Protocol

The participant's traditional socket was scanned using the high-resolution coordinate measurement machine (FaroArm Platinum, FARO Technologies), termed the *original scan*. The shape file was modified using commercial software (Geomagic, Design X, 3D Systems) to create the inner structures as described above, the solid model file was sent for 3D printing and the rest of the fabrication procedure was executed by the research technician. The technician tracked the work time to complete each step of the fabrication process. The socket sensors were calibrated using procedures described in our prior work (31). The fabricated socket was scanned, termed the *pre-session scan*.

Each participant's investigational prosthesis was assembled using the embedded sensor socket, an adjustable pylon, and an energy storage and return prosthetic foot. The initial alignment and componentry positions were the same as the participant's traditional socket (they were included in the shape file). Participants were fitted by the research prosthetist using a traditional dynamic alignment protocol. The alignment set screws were locked using Loctite™, and a torque wrench was used to tighten the rest of the componentry. After fitting was complete, the participant took part in a 30 min test session that involved intermittent 2 min walking bouts separated by sits, stands and partial doffs. The socket was scanned again (termed the *post-session scan*). The participant left the lab wearing the investigational prosthesis.

The participants wore the investigational prosthesis for 4 weeks in their at-home environment. During the first week, the research prosthetist contacted the participant to discuss socket comfort and any issues wearing the investigational socket. If during these discussions the research prosthetist deemed the participant was at risk of injury, she instructed the participant to discontinue use of the investigational prosthesis. Otherwise, after 1 week the participant returned to the lab, the researchers rescanned the socket shape (termed the 1*-week scan*), and the investigational prosthesis was reassembled and returned to the participant. After 4 weeks total take-home testing, the prosthesis was returned to the lab, either in person or by mail, and the researchers rescanned the socket shape (termed the 4*-week scan*). The participant returned to his or her regular prosthesis and left the lab.

Shape comparisons were made in an effort to identify sources of shape change, if and when they occurred. We compared the original scan vs. the pre-session scan; the pre-session scan vs. the post-session scan; the post-session scan vs. the 1-week scan; and the 1-week scan vs. the 4-week scan. The shapes compared were aligned using our custom method for shape coalignment and shape comparison calculations developed in prior work (30). We used an 80:20 volume-to-shape weighting ratio in the algorithm, as used in our prior socket shape assessment research (34). From the patellar tendon to the distal end of the socket, the percent volume differences and the radial errors between the shapes were calculated.

Because some participants commented that the inside surface was rougher than their traditional socket, additional investigational sockets with a vapor-smoothed surface were fabricated. Vapor-smoothing is a process to create a smoother finish on 3D printed plastic parts. Selective laser sintering (SLS), multi-jet fusion (MJF), and fused deposition modeling (FDM) parts are printed with a matte, granular surface finish. For vapor smoothing, the part was placed in a sealed processing chamber with a vapor finishing agent (FA 326 was used on our inner structure material). This vapor melted the surface in a controlled way that evened out the peaks and valleys of the surface. According to the vendor, this resulted in a surface roughness improvement from over 240 *μ*inch Ra to below 125 *μ*inch Ra. The vapor smoothing process was executed such that it maintained the dimensional accuracy of the part.

Bench tests were conducted to characterize the dynamic coefficient of friction between the inner structure and a prosthetic liner that contacted it during clinical use. Three different inner socket materials were assessed: Nyglass, as used in participants' traditional sockets; normal glass-filled blended nylon 12, as used in the first group of investigational sockets; and vapor-smooth glass-filled blended nylon 12, as used in the second group of investigational sockets (described below). The test samples were of dimension 60 mm (W) × 90 mm (L). They were taken from the posterior proximal aspect of the investigational socket above the brimline (Figure 3B). Testing was conducted using a friction tester (Friction Tester, Hanatek) (Figure 9), following procedures described by Cagle et al. (35), which were an extension from the ASTM D1894 “liner friction test” guidelines. The displacement rate was 150 mm/min, the measurement distance was 125 mm and the test weight was 204 g. A section from a silicone elastomeric liner with a fabric backing [70 mm (W) × 210 mm (L)] was used (Alpha Classic, WillowWood). It was preconditioned by leaving it in a 21 °C room for 24 h before testing. The socket samples were mounted to the sled (174.3 g), and the liner was mounted to the bed of the machine fabric side up using double stick tape (FastCap). Six trials were performed on each sample, three in the forward direction and three with the socket samples rotated 180 degrees (reverse direction). The dynamic coefficient of friction was recorded. Means and standard deviations were calculated for each condition.

**Figure 9:**
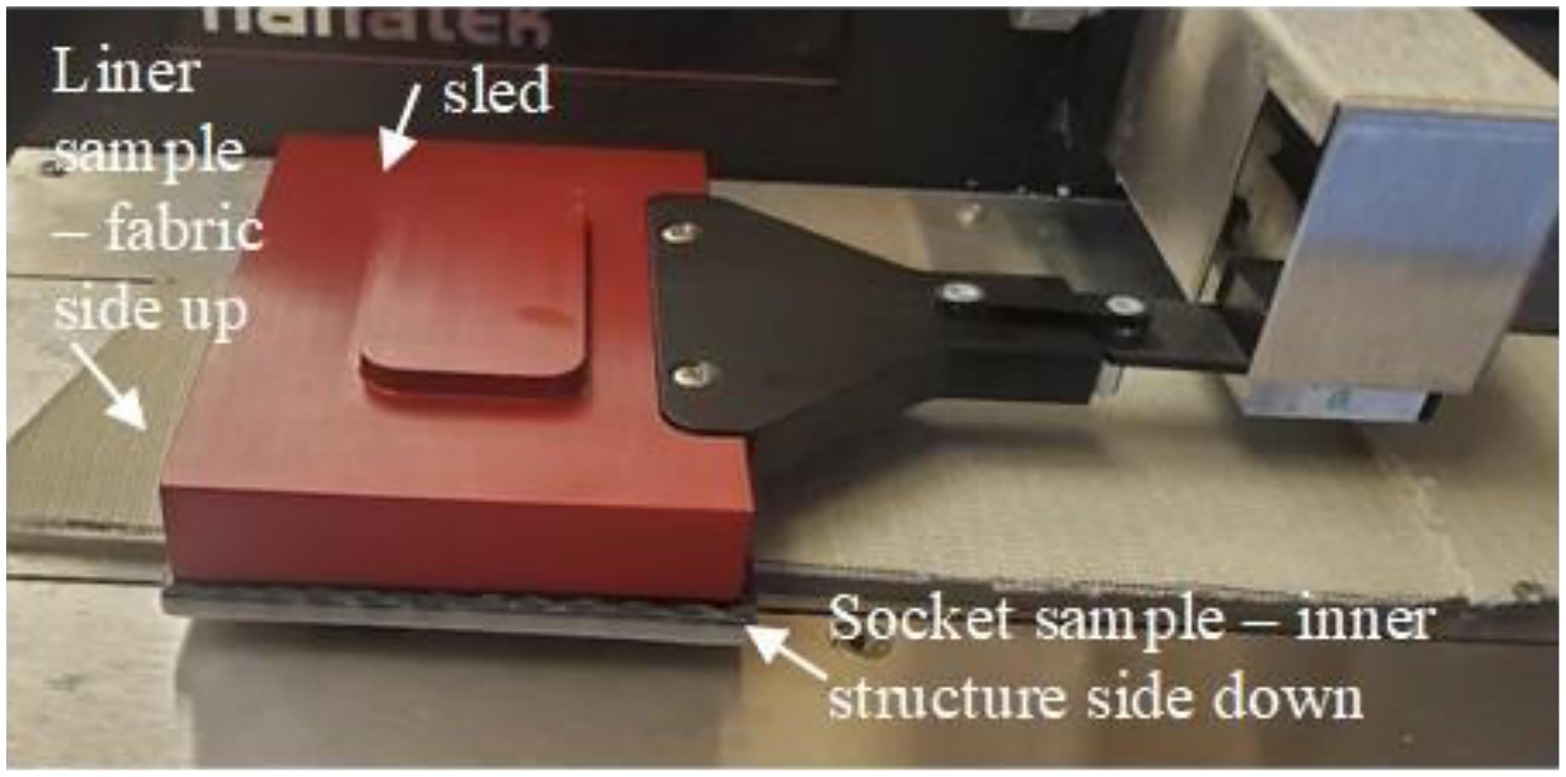
Friction testing. The liner, fabric backing side up, was fastened to the table, and the investigational socket sample (dimension 90 × 60 mm), inner structure side down, was fastened to the sled. The dynamic coefficient of friction, the friction between the inner structure and the fabric backing of the liner, was recorded. The sled was pulled from left to right.

## 3 Results

### 3.1 Bench tests

From the cyclic loading tests on the three short-socket samples conducted in the testing machine, the glass-filled blended nylon 12, the preferred material of the three tested, displayed no meaningful cross-sectional area difference for the original shape compared with the shape of the sample loaded 300,000 cycles (Figure 10A). No indentation or other visible distortion was apparent (Figure 10B), and no powder residue was left on the surface after loading. The volume difference between the original shape and the 300,000-cycle test shape was 0.035%. The median error was −0.0001 mm, and the interquartile range was −0.0441 to 0.0508 mm. The IQR results are outside the industrial scanner's accuracy (0.02 mm) thus there was measurable error, but it was very small and the magnitudes were approximately symmetric about the median. The results were satisfactory, thus the glass-filled blended nylon 12 was used for the inner structure of the investigational sockets, and the two other materials were not considered further.

**Figure 10:**
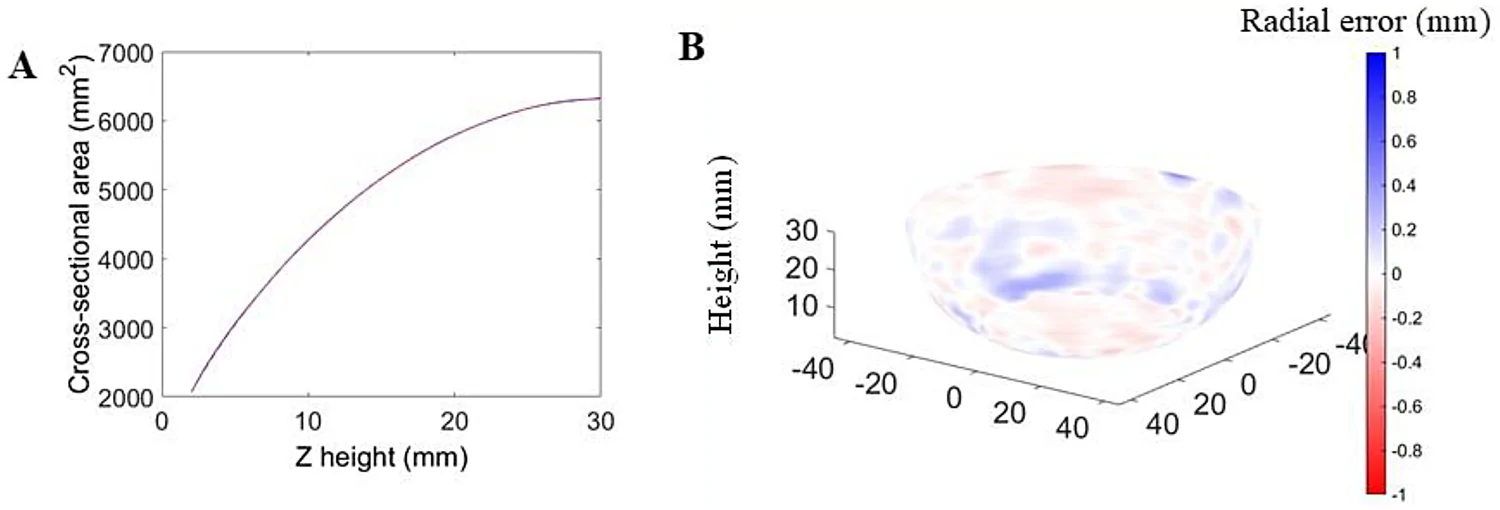
Results from cyclic load testing the glass-filled blended nylon 12 sample shown in Figure 2A. **(A)** Cross-sectional area vs. height plots. Original shape = blue solid line; 300,000 cycle tested shape = red dashed line. The curves were almost identical. **(B)** Radial error difference between the original and 300,000 cycle tested shapes.

### 3.2 Clinical studies

Four participants took part in this study, three males and one female (Table 2). Participants ranged in age from 42 to 65 years. All had their amputation as a result of traumatic injury. All used the investigational prosthesis in western Washington State.

**Table 2:** Participant characteristics.

Partic.	Age (y)	Gdr.	Time since Amp. (y)	Reason for Amp.	Co-Morb.	RL shape	RL Circ. (cm)	RL length- MPT to Distal Tibia (cm)
1	61	M	32	Trauma	HBP	Cylindrical	35.1	11.5
2	65	M	10	Trauma	DM, CHF	Cylindrical	28.2	11.6
3	62	M	16	Trauma	HBP	Conical	27.2	13.3
4	42	F	19	Trauma	None	Bulbous	24.9	14.0

Partic., participant; Gdr., gender; Amp., amputation; Co-Morb., co-morbidities; RL, residual limb; Circ., circumference; MPT, mid-patellar tendon.

During take-home testing, two participants (#1 and #3) commented on the higher surface roughness of the investigational socket compared with their traditional socket (Table 3). One participant (#1) liked this feature because it improved his suspension; the other (#3) disliked it because the investigational socket stuck to the liner fabric and made the socket feel heavier. The socket was of comparable weight to the participant's regular socket. One participant (#2) developed a blister at the patella because the mid-patellar tendon and tibial tuberosity were not well captured in the shape of the inner structure. From our visual inspection of a foam carve of the socket shape compared with the inner structure shape before lamination, this result was due to limitations in the manufacturer's fabrication of the inner structure, not the shape capture method. There was a gap that caused a blister under suction suspension. The participant stopped using the investigational prosthesis on the following day. There was communication with the research prosthetist through rehabilitation to ensure proper and quick healing. Based on the participants' comments, additional investigational sockets with a vapor-smooth finish were fabricated for two participants, #3 and #4. There was no pre-session scan of the vapor smooth socket for participant #3 because of experimental protocol error—the researcher forgot to scan the shape before conducting the in-lab test session. The participant's self-report results for the vapor smooth sockets were more favorable than for the normal material (Table 4), stating that the material on the inside of the socket was the best he had ever used, a happy medium between the normal material configuration (which was too sticky) and his traditional socket (which was too slippery). Participant #4 took longer to engage the locking pin and to settle into the normal-finish socket presumably because of the greater friction from the higher surface roughness.

**Table 3:** Investigational sockets—clinical results.

Partic.	Socket finish	Socket weight (g)	Participant and research prosthetist comments
1	Normal	581	Liked it. The rough surface of the socket helped reduce pistoning during swing phase. Felt improved suspension.
2	Normal	581	Developed a blister at the patella. Because the mid-patellar tendon and the tibial tuberosity geometries were not well captured in the 3-D printed inner structure shape, there was a gap at the patella that caused a blister under suction suspension.
3	Normal	474	Said the socket felt heavy. Prosthetist suspects that the rough surface of the socket made the liner fabric stick, making the distal components feel heavier than the traditional.
	Vapor Smooth	491	The material on the inside of the socket was the best he had ever used, a happy medium between the normal inner-structure configuration (which was too sticky) and his traditional socket (which was too slippery)
4	Normal	478	It took a bit longer to engage the locking pin during the donning process, and it took longer to settle into the socket.
	Vapor Smooth	498	Used the investigational prosthesis every day. No comments on the surface of the socket or difference compared to the regular socket. Participant noted that the prosthesis felt heavier than normal, but this was attributed to the investigational socket weighing more than the participant's regular blade prosthesis, not to the socket interface material.

**Table 4:** Investigational sockets—shape comparison results.

Partic.	Socket finish	Comparison	Volume difference (% socket vol)	Radial error (mm)		
				Median	Q1	Q3
Scanner test	Normal	Scan 1 vs. scan 2	−0.058	−0.011	−0.067	0.037
1	Normal	Original vs. pre-session	−0.418	−0.108	−0.406	0.086
		Pre-session vs. post-session	−0.052	−0.019	−0.093	0.053
		Post-session vs. 1-week	−0.073	−0.033	−0.151	0.084
		1-week vs. 4-week	−0.007	−0.032	−0.125	0.063
2	Normal	Original vs. pre-session	−0.163	−0.079	−0.235	0.076
		Pre-session vs.1-day	−0.067	−0.092	−0.130	0.010
3	Normal	Pre-session vs. 1-week	−0.090	−0.015	−0.108	0.073
3	Vapor	Pre-session vs. 1-week	−0.085	−0.010	−0.130	0.103
		1-week vs. 4-week	−0.093	−0.029	−0.091	0.047
4	Normal	Original vs. pre-session	−0.168	0.009	−0.154	0.155
		Pre-session vs. 2-week	0.089	0.021	−0.024	0.071
4	Vapor	Original vs. 2-week	−0.176	0.061	−0.093	0.192

Computations made for the patellar tendon to the distal end. A number <0 means that the value for the 2nd condition was greater than that for the 1st condition The Scanner test was a repeatability test on the same socket.

Shape comparisons were executed for all available data. The socket shape comparisons showed that the largest volume differences were for the original scan vs. the pre-session socket (Table 4). Investigational socket volume changes reduced over time, suggesting a trend towards a stable shape. Socket volume increases between pre-session and 2 weeks or 4 weeks were all less than 0.18%. For reference, in a prior study we found that an approximately 0.18% or less socket volume change did not cause a clinically detectable change in socket fit (34).

For a technician experienced in making embedded sensor sockets, the total time required to make an embedded sensor socket described above was 88% of that required to make a traditional socket with sensors (730 min vs. 830 min) (Table 5). The embedded sensor socket was completed in 1 day instead of 2 days because in the second part of the process (“Fabrication Step—Part 2” in Table 5) both the Nyglass-resin layup and the lamination needed to be completed instead of just the lamination.

**Table 5:** Technician time and cure time for each step of fabrication.

SOCKET FABRICATION TIME	Technician Time		
Fabrication Steps—Part 1	Traditional Socket	Current Embed. Sensor Socket	
Digitize socket shape using a scanner	50	50	
Traditional socket middle steps			Embed. Sensor Socket middle steps
Process scan to send for fabrication	35	185	Image process socket scan into a digital shape file; add antenna location debosses, connector housing and 4-hole adaptor cutout
Order carve of socket positive	10	5	Order inner structure
TOTAL time (min)	**95**	**240**	
-------------------------Wait 1–3 days for order to arrive-------------			

	Technician Time		Cure Time	
Fabrication Steps—Part 2	Traditional Socket	Current Embed. Sensor Socket	Traditional Socket	Current Embed. Sensor Socket
Do Nyglass/resin layup	60		60	
Fill inner structure with plaster		15		20
Mark antenna wire and plug locations on Nyglass	60			
Prepare antenna wires w/electronics [15 min/antenna, 7 antennae (max)]	105	105		
Integrate harness and place antennae	120	60		
Checks sensors to ensure functionality	10	10		
Place and pot the plug	45	5	30	30
Do outer lamination	90	90	60	60
Trim the brims, grind edges, remove DragonSkin, remove foam/plaster	95	95		
TOTAL time (min)	**585**	**380**	**150**	**110**

Total Time (Technician Time and Cure Time)					
Both Fabrication Steps—Part 1 and Part 2	Traditional Socket	Current Embed. Sensor Socket			
GRAND TOTAL time (min)	**830**	**730**			
Percent time reduction from Traditional Socket	**−**	**12%**			

All times are in minutes. The red text indicates times that may be further reduced by two improvements: adding processing code that automates placing the antenna location debosses, the connector housing and the 4-hole adaptor cutout into the inner structure CAD model; and manufacturing the harnesses rather than custom-making each one.

Bold text indicates total time for each step and for both steps combined.

The dynamic coefficient of friction was significantly higher for the investigational socket made with the normal-finish inner structure compared with the traditional socket (Nyglass-resin layup) (*P* < 0.01) (Figure 11). The mean normal-finish inner structure socket coefficient of friction was 0.305 (±0.033) and the traditional socket was 0.203 (±0.009). Results between the regular and reverse-positioned tests were comparable, suggesting minimal history dependence to the direction of applied load. The vapor-smooth-finish inner-structure socket had a dynamic coefficient of friction 0.200 (±0.006) significantly lower than the normal-finish inner-structure socket (*P* < 0.01) and not significantly different from a traditional socket (*P* = 0.89).

**Figure 11:**
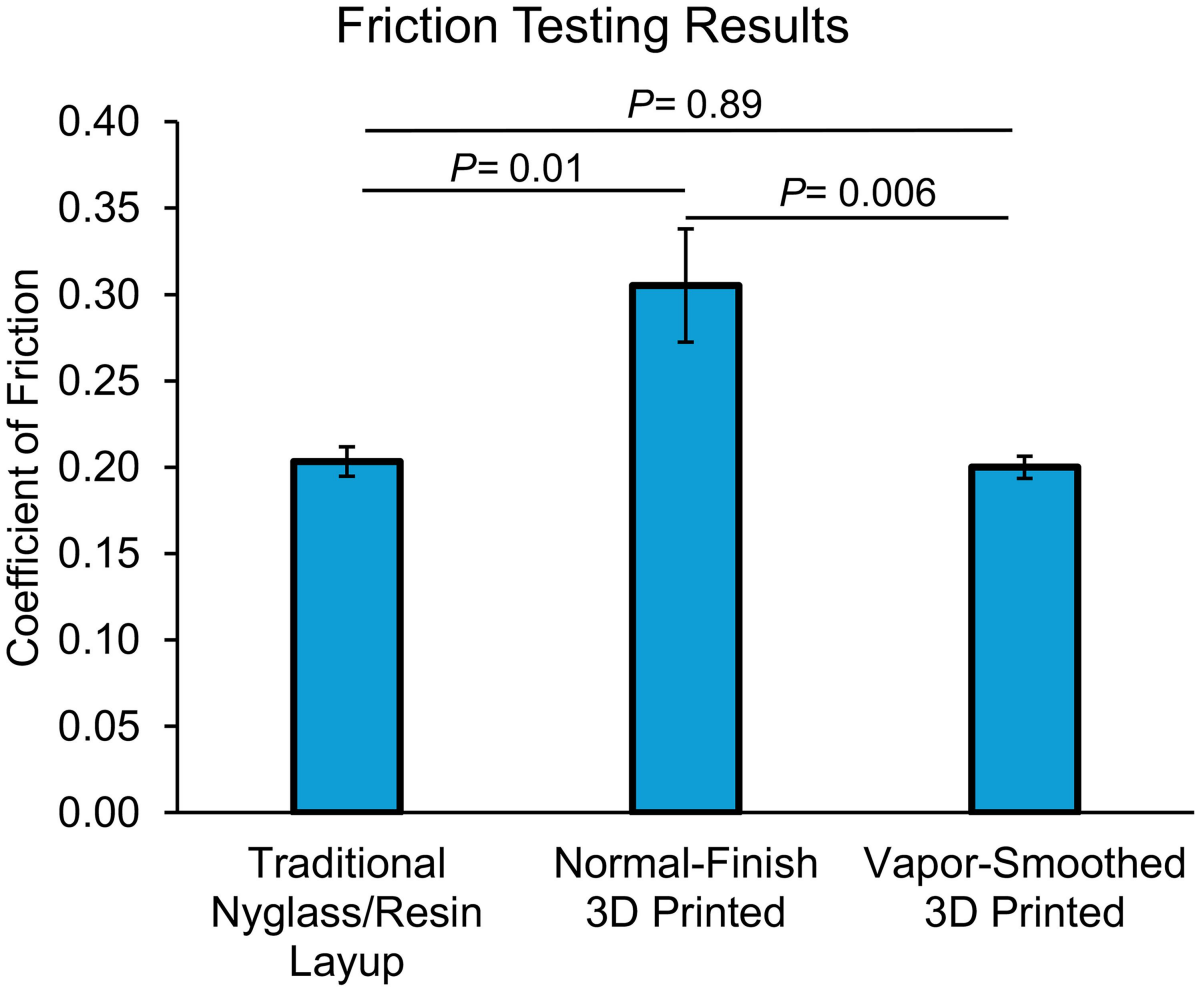
Dynamic frictional coefficient results. Traditional sockets (traditional Nyglass/resin layup) are compared with normal-finish, 3D printed, inner-structure sockets and vapor smooth-finish, 3D printed, inner-structure sockets. Means and standard deviations are shown.

## 4 Discussion

The lack of a time-efficient and cost-effective strategy for embedding sensors into prosthetic sockets in a manner that maintains their function during patient at-home use has been a barrier to the integration of interface sensor data into clinical care (5–8, 11, 12, 16). Previously reported strategies altered the inside socket shape, required excessive clinical time to mount, were excessively heavy or bulky or were prone to damage and thus were costly to maintain. Some interface pressure systems have been used in commercially available shoe pressure monitoring systems (e.g., Orpyx) but applying these technologies to prosthetic sockets may introduce challenges. If a time-efficient and cost-effective strategy for embedding sensors into prosthetic sockets were achieved, clinical and computational science researchers specializing in outcome assessment could investigate which quantitative metrics help prosthetists meaningfully enhance their patients' clinical care. Machine learning strategies may be useful. Embedded sensor sockets could be regularly used in patient free-living environments, paving the way for research databases to promote collaborative discovery towards effective clinical use of sensor data, an enhanced quality of life for our prosthesis user patients and quantitative justification for reimbursement.

The strategy described in this paper improved upon the state-of-the-art by creating a technology that simplified fabrication of an embedded sensor socket and required 12% less time than making a traditional embedded sensor socket. The process was less time-consuming because the 3D-printed inner structure replaced the first lamination (Nyglass-resin layup) and the marking of antenna wire and plug locations on the Nyglass, which took about 120 min (Table 5). Further, several components of the sensing system previously attached one at a time (antennae, electronic components, wires, connectors) (11) were now assembled *a priori* as an easy-to-install single unit, saving 60 min. A next advance that will meaningfully further reduce technician time is to automate the inner structure design process, i.e., *Embedded Sensor Socket middle steps* in Table 5. By adding processing code that automates placing the antenna location debosses, the connector housing and the 4-hole adaptor cutout into the CAD model, technician time may be reduced from the 185 min shown in Table 5 to possibly as little as 10 min. Vendor manufactured harnesses of different sizes may reduce the 105 min time currently required to prepare the antennae, wires and electronics to approximately 5 min. The 60 min time to integrate the harness and place antennae required using the current method may be reduced to 15 min. A quick-and-easy-sensor-check instrument may reduce the time to check the sensors to ensure functionality from 10 min to 5 min. Altogether, these enhancements may decrease the technician time to make an embedded socket to 405 min, a 51% reduction from the traditional embedded sensor socket. These estimates are conjecture and would need to be validated through rigorous scientific investigation. Interested stakeholders, e.g., prosthetists and clinical researchers, may be able to quickly manufacture in their clinic or purchase from a central fabrication facility an embedded sensor socket ready to plug in and use. In the future prosthesis users or caregivers with 3D printers at home may be able to manufacture patient prosthetic sockets with embedded sensors.

The result that the inner structure material showed no meaningful deformation under cyclic loading (300,000 cycles is typically about a third of the steps taken by a prosthesis user per year) was an important milestone because it suggested that at least some current 3D printed materials were of adequate strength for this application. Our prior efforts integrating sensors into custom inserts placed into sockets (13, 36) showed settling during up to 4 weeks of take-home use. Long-term testing in participants' free-living environments is needed to determine if clinically meaningful deformation or mechanical failure of the inner structure occurs before the expected socket life is reached. New 3D printed materials with improved strength will likely emerge over time, potentially enhancing the material choices for the inner structure. Though the weights of the sockets in the present study were comparable to participants' traditional sockets, in the future a more lightweight inner structure material and thus a lighter prosthesis may enhance outcomes.

Results from this study showed that the most meaningful shape error, which was between the original socket shape file and the pre-session, ranged from −0.163% to −0.418% socket volume error. Results reported in the literature on transtibial prosthesis user participants demonstrated that a volume error greater than 1.00% caused clinically detectable changes in socket fit (34). The original scan vs. the pre-session socket volume difference reflected a combination of the fabrication error in the 3D-printing executed by the vendor, deformation of the inner structure from the lamination and deformation during the first hours of use. Our study did not allow us to delineate the relative influence of the three variables. Vendors who fabricated the 3D printed inner structures in the present study estimated accuracies of ±0.25 mm (add 0.02 mm per cm of height). This error would account for all of the volume error between original scan vs. pre-session socket reported in Table 4. However, the lamination process which involves pouring hot resin over the outside of the inner structure would be expected to compress the inner structure and increase socket volume, consistent with the results shown in Table 4. Further investigation delineating 3D printing error from lamination and continual mechanical loading effects would help focus future research needs if socket shape accuracy proves problematic in clinical research investigations. Though subsequent shape comparisons over the following 4 weeks were not available for some of the participants (because of participant scheduling challenges), the results we did obtain were consistent with the interpretation that the changes were asymptotic and reduced over time. We suspect that Participant #2's blister formed primarily because the 3D printed inner-structure did not well-capture his traditional socket mid-patellar tendon and tibial tuberosity geometries. The error was visually evident in the inner structure received from the vendor. Fidelity issues in 3D printing have improved since this research was conducted and continue to improve. Shape error issues are thus less likely to occur and impact socket fit.

The inner structure surface roughness in this study was an important variable to participant comfort. The 0.1 coefficient of friction difference between 3D printed sockets and vapor-smoothed 3D printed sockets was significant (*P* = 0.006), suggesting that even small changes in surface roughness may have a meaningful clinical impact. This result is consistent with work by other researchers. Quinlan et al. (37, 38) using a bench test apparatus showed a statistically meaningful difference in suspension when the texture of the socket surface was changed. Further, finite element models that modeled the liner-socket interface as a frictional interface showed strong matches between areas of high interface pressure and shear stress and skin breakdown 6 months later (39), demonstrating the importance of the presence of a frictional liner-socket interface to interface mechanics and ultimately soft tissue health. As suggested by others (37, 38), the surface roughness may be tuned to the prosthesis user's socket fit needs.

A forerunner of non-contact sensing in 3D printed sockets is limb motion sensing. Inductive limb-motion sensors were used as an example in this research. As stated in laboratory gait analysis studies in the literature (12–16, 40, 41), distance sensing may introduce a new array of sensed variables for potential use in biomechanical analysis, outcome assessment, prosthetic design and fitting and other areas relevant to patient care. We have used distance sensing to create a detailed characterization of activity (42, 43), to determine if the prosthesis was donned (42), to detect day-to-day differences in suspension (13) and for feedback control of an auto-adjusting socket that improved the stability of a socket fit metric (31, 44, 45). Potentially, the methods described in the present research could be extended and used for other non-contact sensing technologies. A robust array of embedded socket sensor choices may enhance information available to practitioners to educate and treat their patients to improve outcomes and enhance their quality of life.

## 5 Conclusion

The time-efficient fabrication process and the strong mechanical and clinical test results shown in this study indicated that we overcame the barriers in traditional socket fabrication that previously prevented the easy integration of limb motion sensors into prosthetic sockets. The findings warrant implementation of the embedded sensor sockets in take-home clinical trial investigations to pursue two next steps in the research: long-term assessment of time-dependent deformation; identification of quantitative metrics detected by the sensors that help prosthetists meaningfully enhance their patients' quality of life.

## Data Availability

The original contributions presented in the study are included in the article/Supplementary Material, further inquiries can be directed to the corresponding author/s.
